# Prevalence of financial and social challenges and their associations with major satisfaction in animal and dairy science undergraduates

**DOI:** 10.1093/jas/skag093

**Published:** 2026-04-01

**Authors:** Jennifer M Bundy, Dariana Glasco-Boyd, Justin J Roberson, Howard D Tyler, Jessica Wikstrom

**Affiliations:** Department of Animal Science, Iowa State University, Ames, IA 50011, United States; Undergraduate Business Career Services, Ivy College of Business, Iowa State University, Ames, IA 50011, United States; Des Moines Area Community College, Des Moines, IA 50314, United States; Department of Animal Science, Iowa State University, Ames, IA 50011, United States; Office of Academic Innovation and Student Success, College of Agriculture and Life Sciences, Iowa State University, Ames, IA 50011, United States

**Keywords:** financial stress, peer relationships, first-generation students, transfer students, student success, retention

## Abstract

Student success is shaped by both financial stability and social connectedness, yet little research has examined these dynamics within Animal and Dairy Science programs. This study investigated financial well-being, peer relationships, and academic and career satisfaction among 298 undergraduates in Animal Science (ANS), Dairy Science (DYS), and General Pre-Veterinary (GENPV) at a large land-grant university (33% response rate). A significant GPA difference of 0.45 points was observed between respondents and non-respondents (3.47 ± 0.52 vs. 3.02 ± 0.84; *P* **<** 0.01), suggesting possible non-response bias. Some financial strain was reported: 11% of students frequently worried about basic living costs and 12.5% about academic expenses. First-generation students were more likely to worry about tuition and fees (*P* **<** 0.01) and reported less family support for academic (43% vs. 30%; *P* **<** 0.01) and living expenses (51% vs. 38%; *P* = 0.04) than their peers. Transfer students also reported reduced financial support (58% vs. 30% of direct-from-high school; *P* **<** 0.01). Social connections were generally strong, yet transfer students were more likely to lack close friendships (16% vs. 8%; *P* = 0.02) and reported lower satisfaction with peer relationships (14% vs. **<**5%; *P* **<** 0.01). Lack of close peer support was linked to lower GPA (3.23 ± 0.66 vs. 3.48 ± 0.48; *P* = 0.05). Academic satisfaction was high overall (**>**88%), but transfer students reported greater dissatisfaction with intellectual development (10% vs. 3%; *P* **<** 0.01) and academic experiences (16% vs. 5%; *P* = 0.03). Career awareness was also high (**>**85%), though transfer and students of color reported significantly lower familiarity (*P* = 0.02 and *P* = 0.05, respectively). Dissatisfaction with career opportunities was associated with lower GPA (3.38 ± 0.51 vs. 3.53 ± 0.47; *P* **<** 0.01). These findings highlight the disproportionate challenges faced by transfer and first-generation students, whose financial insecurity and weaker peer networks contribute to lower satisfaction and achievement. Addressing these disparities requires targeted support in financial aid, peer integration, and career mentoring to foster equitable student success in agricultural sciences.

## Teaser

This study reveals how financial pressures and peer connections shape the experiences of Animal and Dairy Science undergraduates, with first-generation and transfer students facing the greatest challenges.

## Introduction

Student success in higher education is shaped by a complex interplay of financial, social, and academic factors that extend beyond classroom instruction alone. Decades of research demonstrate that traditional academic indicators such as standardized test scores and prior GPA explain only a portion of the variance in college outcomes, while non-cognitive and contextual factors play a substantial and often underexamined role (Robbins et al. 2004; Sparkman et al. 2012). As institutions seek to improve retention, satisfaction, and timely degree completion, increasing attention has been directed toward students’ financial well-being and social integration as critical contributors to academic persistence and achievement.

Financial stress is consistently identified as a major barrier to student success. Students experiencing financial strain are more likely to report poorer academic performance, elevated stress, reduced course loads, and increased likelihood of stopping out or leaving college altogether (Dennis et al. 2005; Smathers et al. 2024). These pressures are not evenly distributed across student populations. First-generation and transfer students, in particular, are more likely to receive limited familial financial support and to shoulder a greater share of educational and living expenses, placing them at heightened risk for academic and psychological strain (Harvey-Smith 2002; Almeida et al. 2021). Importantly, perceived financial stress, rather than objective cost alone, has been shown to meaningfully influence students’ educational decision-making and persistence behaviors (Smathers et al. 2024), underscoring the value of examining students’ own financial experiences.

Alongside financial considerations, social connectedness and peer relationships are central to student adjustment and success. Foundational models of student persistence emphasize that academic outcomes are strongly influenced by students’ integration into the social and intellectual life of the institution (Tinto 1993; Tinto and Pusser 2006). Empirical evidence further demonstrates that peer relationships contribute to a sense of belonging, academic motivation, emotional support, and intellectual engagement (Pedler et al. 2022; Hoyt 2023). Recent work suggests that the quality of peer relationships may be more strongly associated with academic performance than relationships with parents or instructors, particularly during the undergraduate years (Yu et al. 2023). Conversely, students who lack close peer connections are more likely to experience social isolation, lower satisfaction, and weaker academic outcomes (McCabe 2023).

These dynamics are especially important for student populations navigating transitional or marginalized positions within higher education. Transfer students often experience “transfer shock,” characterized by reduced academic performance and social disconnection following matriculation, particularly when peer support networks are weak (Cepeda et al. 2021). First-generation students similarly report lower levels of belonging and social capital, which can undermine both academic confidence and persistence (Dennis et al. 2005; Almeida et al. 2021; Pedler et al. 2022). Social Cognitive Theory provides a useful framework for understanding these patterns, emphasizing that student success emerges from the interaction of personal agency, proxy support (e.g., advisors and instructors), and collective or environmental agency, including peer networks (Bandura 2002). When collective support systems are limited, students’ ability to navigate academic and career pathways may be compromised.

Despite a robust body of literature documenting the importance of financial stability and social connectedness across higher education broadly, relatively little work has examined how these factors manifest within agriculture-based majors. Animal Science and Dairy Science programs encompass diverse curricular pathways, experiential learning requirements, and career trajectories, yet students within these disciplines may face unique financial pressures related to laboratory courses, unpaid experiential learning, and extended time-to-degree. At the same time, the cohort-based nature of many Animal Science programs may shape peer relationships in ways that differ from other academic contexts. To date, there is limited empirical evidence describing the prevalence of financial concerns, peer connectedness, and satisfaction with academic and career preparation among students in these majors.

Accordingly, the purpose of this exploratory study was to characterize the prevalence of financial concerns, the extent of peer relationships, and levels of satisfaction with the major and career pathways among undergraduate students in Animal and Dairy Science. By examining these experiences across key demographic groups, this study seeks to establish a foundational understanding of the financial and social landscapes facing students in this discipline and to identify areas warranting deeper, multivariable investigation in future research.

## Materials and methods

This study was reviewed and approved as exempted research by [UNIVERSITY] Institutional Review Board (IRB: 23-410) for Human Subjects Research and complied with CFR 45, Part 46. This report focuses on a subset of data from a larger, college-wide study.

### Survey instrument

The research team identified key factors for investigation and conducted a review of existing survey instruments to determine whether a new instrument was necessary. Pascarella and Terenzini (1980) developed and validated a survey instrument comprising four question sections: (I) Peer-Group Interactions, (II) Interactions with Faculty, (III) Faculty Concern for Student Development and Teaching, and (IV) Academic and Intellectual Development. These questions encompassed most of the intended topics. However, they did not include measures of financial well-being or academic advisor interactions. To address these gaps, the research team adapted the existing survey by incorporating two additional sections: Interactions with Academic Advisors and Financial Well-Being. For this report, we focus on the prevalence of financial concerns, the extent of peer relationships, and student satisfaction within the major, without modeling the interconnectedness of all factors collected. Accordingly, faculty and advisor interaction variables are not reported here.

The survey instrument was administered through the Qualtrics online survey platform (Version 2024; Provo, UT, USA) and consisted of 32 questions. Participation was voluntary, and respondents were required to provide informed consent, allowing the research team to access de-identified academic and demographic data through registrar records. There were no penalties for non-participation, and the survey was not linked to any specific course or program. To encourage participation, an incentive was offered in the form of a random drawing for twenty $20 Visa gift cards and two $75 Visa gift cards. Non-respondents received two reminder emails over a three-week period.

### Validity and reliability of the modified survey instrument

Confirmatory factor analysis (CFA) was conducted to evaluate the construct validity of the modified survey instrument, which was based on the previously published tool (Pascarella and Terenzini 1980) with additional items included. The CFA results indicated acceptable-to-strong model fit, as evidenced by a Tucker-Lewis Index (TLI) of .934 and a Root Mean Square Error of Approximation (RMSEA) of .054. A TLI value above .90 suggests good comparative fit relative to a baseline model, while an RMSEA value below .06 indicates a close approximation of the model to the observed data. These values collectively support the adequacy of the hypothesized factor structure. All standardized factor loadings were above .60, indicating that individual survey items demonstrated strong relationships with their respective latent constructs. This level of loading provides evidence that the items meaningfully represent the theoretical factors they were intended to measure, further supporting the construct validity of the instrument. The internal consistency of each scale was examined using Cronbach’s alpha. Values ranged from .74 to .95, exceeding the commonly accepted threshold of .70 for acceptable reliability. These results suggest that the survey scales demonstrated strong internal consistency, with individual items within each scale consistently reflecting the underlying construct. Together, the CFA and reliability analyses provide support for the use of the modified instrument in future research.

### Eligible participants and non-response error

The survey instrument was distributed via email to all undergraduate students within the agriculture division at Iowa State University inviting a total of 3,466 students to participate. Upon survey closure, identifiable student responses were sent to the registrar’s office, where academic and demographic data were matched to the survey data. The dataset was then deidentified and returned to the research team. For this report, we extracted data from respondents in the Animal Science (ANS) and Dairy Science (DYS) majors, and students that have declared the General Pre-Veterinary Option. The General Pre-Veterinary Option is not a major. It is an option that can be added to a degree program such as Animal or Dairy Science. However, if a student has not declared a major, but has expressed interest in the Pre-Veterinary path, they can be listed as solely General Pre-Veterinary Medicine. Both majors and the Pre-Veterinary option are housed within the Department of Animal Science. To assess potential non-response bias, an Analysis of Variance (ANOVA) was conducted to compare the grade point averages (GPA) of respondents and non-respondents from the ANS, DYS, and GENPV cohorts.

### Survey responses and testing for demographic differences

Survey responses were logged and tallied. A linear model was used to test the fixed effects of classification year, admission type, sex, first-generation status, ethnicity code, and cumulative GPA on the categorical response of each question. All fixed effects were based on admission records provided by the university registrar. Classification year included four levels: freshmen, sophomore, junior, senior. However, many incoming students have previously earned credit through dual enrollment at a community college or advanced placement exams, so that a small proportion of new students come in as true freshmen (less than 30 credits). Admission type includes two levels: direct-from-high school (DFH) and transfer students. Two levels of sex and first-generation status were used: females and males, and yes or no, respectively. Eight levels of ethnicity were used: Alaskan or Native American, Asian, Black, White, Hispanic, Hawaiian or Pacific Islander, two or more races, or prefer not to answer. Cumulative GPA was given in a continuous format from 0.00 to 4.00. If a fixed effect was determined to be significant with a *P* < 0.05, then a chi-square test was used to test the significance of the categorical fixed effects on the answers given. In the case of GPA, an ANOVA was used to compare average GPAs across categorical responses or across categorical demographics. If a Chi-square or ANOVA test revealed a *P*-value less than 0.05, the difference was deemed as significant.

## Results

### Non-response error

Across all agricultural majors, a total of 679 students participated in the study out of the 3,466 that were invited to complete the survey (19.59% participation rate). However, for the subset of respondents reported here, there were 891 ANS, DYS, and GENPV students invited to participate and 298 respondents (33% response rate). Although the response rate was satisfactory, non-response bias remains a concern. Previous research indicates that students who are struggling academically or feel disengaged are less likely to complete surveys, potentially skewing results (Groves and Peytcheva 2008). Within this subset of data, a significant difference of 0.45 GPA points was observed between the average cumulative GPA of respondents and non-respondents (*P* < 0.01). Means and standard deviations of GPA among respondents and non-respondents are provided in Table 1.

**Table 1:** Analysis of non-response error.

Student group	**GPA** ^1^ **Mean**	GPA Std. Dev.	ANOVA	
**Non-respondents**	3.02	0.84	*F*-Ratio	*P*-Value
**Respondents**	3.47	0.52	72.11	<0.01

1 Grade Point Average (GPA) is based on a 4.00 scale.

### Participant demographics

Participant demographics across major or option, admission type, sex, classification year, first-generation status, and ethnicity are provided in Table 2. The vast majority of students declared the ANS major (*n* = 284), with very few DYS majors (*n* = 10) or GENPV (*n* = 4). This ratio is similar to the percentages of ANS, DYS, and GENPV students across the departmental population. Over 80% of respondents were direct-from-high school, which falls in line with the departmental distribution of students. Just over 90% of respondents were female, which is only slightly higher than the current gender distribution across animal-related majors and disciplines. Over 42% of the participants were classified as seniors compared to just over 9% that were classified as freshman. As discussed above, the classification year tends to be skewed due to transfer credits, advanced placement credits, and dual enrollment courses completed in high school. Therefore, classification year may not be the best metric to use in future analyses. Interestingly, just over 30% of respondents were first-generation students when first-generation student makes up only 10% of the departmental population. There were 90 first-generation students that participated out of 362 that were invited to participate, which is a first-generation response rate of over 44%. Unfortunately, the college is not a very diverse academic unit as just over 88% of respondents were White. For statistical analysis, students of color were pooled and consisted of Black (*n* = 1), and Hispanic (*n* = 21).

**Table 2:** Demographics of survey respondents.

	*N*	% of total
**Declared major or option** ^1^		
**Animal Science**	284	95.3
**Dairy Science**	10	3.4
**General Pre-Veterinary Med.**	4	1.3
**Admission type**		
**Direct from high school**	246	82.6
**Transfer**	52	17.4
**Sex**		
**Female**	269	90.3
**Male**	29	9.7
**Classification** ^2^		
**Freshman**	27	9.1
**Sophomore**	60	20.1
**Junior**	84	28.2
**Senior**	127	42.6
**First generation status**		
**No**	208	69.8
**Yes**	90	30.2
**Ethnicity** ^3^		
**White**	265	88.9
**Students of color**	22	7.4
**More than one race**	5	1.7
**Prefer not to answer**	6	2.0

1 General Pre-Veterinary Medicine is not a major. It is an option that can be added to a degree program such as Animal or Dairy Science. However, if a student has not declared a major, but has expressed interest in the Pre-Veterinary path, they can be listed as solely General Pre-Veterinary Medicine and are housed within the Department of Animal Science.

2 Classification is determined by the number of credits that a student has earned. It is possible for a first-year, direct-from-high school student to be classified as a sophomore or above.

3 For statistical analysis, students of color were pooled. This group consisted of African American (*n* = 1), and Hispanic (*n* = 21) students, based on the registrar 10th day registration list.

### Participant responses—financial well-being

Responses related to the perception of financial well-being are provided in Table 3. A noticeable portion of Animal Science students reported financial strain during their academic experience. Approximately 11% of students indicated frequent concern about meeting basic living expenses (e.g., food, rent), and 12.5% reported similar levels of concern regarding academic-related costs such as tuition and supplies. Financial anxiety was more pronounced among first-generation college students, who were significantly more likely than their non-first-generation peers to worry about academic expenses (*P* < 0.01). When asked whether financial constraints prevented participation in recreational activities, just over 13% of respondents indicated that cost had been a barrier. However, no significant demographic differences were observed for this item.

**Table 3:** Perception of financial stability of animal science students.

Question How often do you…	Responses (%)				
	Never	Sometimes	About half the time	Most of the time	Always
**worry about being able to meet normal monthly living expenses?**	39.1	37.6	12.5	6.6	4.2
**worry about being able to cover academic expenses?**	42.7	36.7	8.1	7.3	5.2
**want to take part in recreational activities, but can’t afford to?**	25.8	46.0	15.0	9.7	3.5

Family financial support is represented in Table 4., and coverage of expenses varied widely across the student population. About 35% of students stated that none of their academic expenses were covered by family or legal guardians. This proportion was notably higher among first-generation students (43% vs. 30% of non-first-generation; *P* < 0.001) and transfer students (58% vs. 30% of direct-from-high school (DFHS) students; *P* < 0.01). In contrast, only 8% of first-generation and 9.6% of transfer students reported that all academic expenses were covered, compared to 30% of non-first-generation and 26% of DFHS students, respectively.

**Table 4:** Familial financial support of animal science students.

Question	Responses (%)		
	None	Some	All
**___ of my academic expenses are covered by family or guardian**	35.2	40.7	24.1
**___ of my living expenses are covered by family or guardian**	42.9	44.6	12.5

Similar patterns emerged for coverage of living expenses. Nearly 43% of all respondents reported that none of their living expenses were covered by family or legal guardians. First-generation (51%) and transfer students (60%) were significantly more likely to report no familial support for living expenses than their non-first-generation (38%; *P* = 0.04) and DFHS peers (38%; *P* = 0.05), respectively. Only 6% of first-generation and 8% of transfer students reported full familial support for living expenses, compared to 14% and 13% of non-first-generation and DFHS students, respectively.

### Social connectedness and peer relationships

The perception of peer relationships across all respondents is provided in Table 5. While most students felt positively about their peer relationships, key differences emerged by admission type and first-generation status. Transfer students were more likely to report a lack of close friendships (16%) compared to DFHS students (8%; *P* = 0.02). Similarly, 13% of first-generation students reported difficulty forming satisfying relationships, compared to 8% of their peers (*P* = 0.07). GPA was associated with perceived social support, as students who reported lacking close peer relationships had a lower average GPA (3.23 +/- 0.66) than those who did (3.48 +/− 0.48; *P* = 0.05).

**Table 5:** Perceived peer connections among animal science students.

Rank your level of agreement with each statement:	Responses (%)				
	Strongly disagree	Somewhat disagree	Neither agree nor disagree	Somewhat agree	Strongly agree
**Since coming to this university, I have developed close personal relationships with other students**	0.8	7.7	6.1	28.3	57.1
**The student friendships I have developed at this university have been personally satisfying.**	1.2	4.9	8.5	24.3	61.1
**My interpersonal relationships with other students have had a positive influence on my intellectual growth.**	1.2	1.6	13.4	30.8	53.0
**I have at least one friend at this university that would be willing to help me if I had a problem.**	1.2	2.1	2.4	17.4	76.9
**Many students at this university have values that are different than mine.**	2.4	8.9	23.9	38.1	26.7

When asked about the quality of their friendships, 14% of transfer students reported dissatisfaction compared to less than 5% of DFHS students (*P* < 0.01). Additionally, over 14% of transfers felt that peer relationships had not positively influenced their intellectual growth, compared to fewer than 2% of DFHS students (*P* < 0.01). A small proportion of upper-level students (5% of juniors and seniors) reported the same, while no freshmen or sophomores did (*P* = 0.03). Regarding emotional support, nearly 6% of transfer students and 11% of freshmen reported lacking a friend they could rely on during a problem, compared to only 2.6% of DFHS students and fewer than 2% of students in other classifications (*P* < 0.01 and *P* = 0.01, respectively).

### Academic satisfaction and perceived growth

Overall, students reported high levels of academic satisfaction and intellectual development. More than 89% expressed satisfaction with their intellectual development, and nearly 88% felt that their academic experiences had a positive influence on their growth. However, transfer students were significantly more likely to report dissatisfaction with their intellectual development (10% vs. 3% of DFHS students; *P* < 0.01) and academic experience (16% vs. 5%; *P* = 0.03). Although the observed differences did not reach statistical significance, First-generation students were also more likely to report dissatisfaction (11% vs. 5%; *P* = 0.07), and their average GPA was lower among those dissatisfied (3.28 ± 0.58) compared to those satisfied (3.51 ± 0.47; *P* = 0.07). Similarly, 14% of transfer students reported that their coursework was not intellectually stimulating, compared to just 3% of DFHS students (*P* = 0.08). Seniors were also more likely to feel unstimulated by their coursework than underclassmen (*P* = 0.07).

### Career awareness and satisfaction

A majority of students (88%) indicated familiarity with career opportunities related to their major. However, 11% of transfer students reported unfamiliarity compared to less than 5% of DFHS students (*P* = 0.02). Ethnicity also played a role, with nearly 13% of students of color students feeling unfamiliar with career opportunities, compared to about 4% of White students (*P* = 0.05). Satisfaction with career opportunities mirrored these findings. Over 85% of respondents were satisfied, though nearly 11% of transfer students and 11% of first-generation students expressed dissatisfaction (*P* = 0.02 and *P* = 0.07, respectively). GPA was again linked with perception such that students who were dissatisfied with career opportunities had a significantly lower average GPA (3.38 ± 0.51) compared to those who were satisfied (3.53 ± 0.47; *P* < 0.01).

## Discussion

This study explored the financial and social well-being of undergraduate students within the Animal Science discipline, revealing distinct challenges related to financial insecurity and social integration, particularly among transfer and first-generation students. These findings align with and expand upon existing research on student success, highlighting the need for targeted institutional action to support vulnerable student populations.

### Financial constraints and educational equity

A significant proportion of students reported frequent worry about living and academic expenses, with first-generation and transfer students disproportionately impacted. These findings echo Harvey-Smith (2002) model of minority student retention, which identifies finances as a central barrier to persistence. Similarly, Dennis et al. (2005) found that lack of family financial support among first-generation students significantly hindered GPA and commitment to college, mirroring our finding that a sizable share of these students receive no support for tuition, books, or living costs.

The disparities between first-generation and continuing-generation students are especially important in light of Bandura (2002) Social Cognitive Theory, which emphasizes the interdependence of personal agency and collective support systems. When family-based financial support is lacking, institutional support structures must compensate through advising, aid, and access to affordable academic and co-curricular experiences. These efforts are essential to ensure equitable opportunities for success.

### Social integration and peer relationships

The importance of peer connection has emerged as another noticeable theme. Transfer students, in particular, reported weaker social bonds, fewer close friendships, and less satisfaction with peer relationships. These social gaps correlated with lower GPA and lower perceived intellectual development, consistent with the concept of transfer shock and research by Cepeda et al. (2021), who found that transfer students with limited social support struggled academically. Tinto and Pusser (2006) also emphasized that persistence is heavily influenced by students’ sense of belonging and peer integration. Both of these factors that appear lacking among many transfer students in our sample.

First-generation students similarly reported difficulties forming strong peer relationships. Almeida et al. (2021) highlights that among first-generation students, social capital is a stronger predictor of academic success than grit. This underscores the importance of fostering meaningful connections early and intentionally, especially for those whose backgrounds may not naturally provide those networks. Furthermore, our findings that social connectedness is associated with GPA and intellectual satisfaction support the work of DeBerard et al. (2004) and Robbins et al. (2004), both of which found that social support, peer engagement, and psychosocial well-being are predictive of college GPA. Addressing these needs is especially pressing, as the effects of isolation extend beyond academic performance to impact emotional resilience, adjustment, and long-term persistence (Sharma and Yukhymenko-Lescroart 2024).

### Academic and career satisfaction

Despite financial and social barriers, a large majority of students expressed satisfaction with their intellectual development and academic experience, as shown in Table 6. Yet, dissatisfaction was again more common among transfer and first-generation students which are groups who also reported lower familiarity and satisfaction with career opportunities. These findings align with previous research (Parnes et al. 2020; Harris and Nagle 2023) showing that underrepresented students benefit significantly from close faculty relationships and mentoring when it comes to career clarity and intellectual confidence. Without such relationships, students may remain unaware of the full range of professional opportunities available to them, undermining long-term success.

**Table 6:** Major and career satisfaction of animal science students.

Rank your level of agreement with each statement:	Responses (%)				
	Strongly disagree	Somewhat disagree	Neither agree nor disagree	Somewhat agree	Strongly agree
**I am satisfied with the extent of my intellectual development since enrolling in my major.**	1.8	2.5	6.1	41.8	47.8
**My academic experience had had a positive influence on my intellectual major.**	1.1	4.7	4.7	35.5	54.0
**I am satisfied with my academic experience within my degree program.**	1.5	5.4	5.4	37.7	50.0
**The courses within my major are intellectually stimulating.**	1.8	3.6	5.1	37.5	52.0
**I am familiar with career opportunities associated with my degree program.**	0.7	4.8	6.6	38.8	49.1
**I am familiar with career opportunities associated with my degree program.**	1.1	4.7	9.0	31.4	53.8

The observed associations between GPA and satisfaction with academic and career experiences also resonate with the meta-analysis by Robbins et al. (2004), which identified academic self-efficacy and motivation as strong predictors of achievement. Students who are more engaged with their curriculum, and who see clear professional pathways, are more likely to remain motivated and perform well academically.

### Limitations of the data

The current dataset has several important limitations that should be considered when interpreting the results and applying them to broader student populations. The demographic makeup of the sample limits the scope of its application. With over 88% of respondents identifying as White, it is difficult to draw meaningful conclusions about students from other racial or ethnic backgrounds. Academic classification was also based on the number of credits earned, including dual enrollment and AP credits, which resulted in many first-year students being categorized as sophomores. Because only a small number of true freshmen responded, interpretations about first-year student experiences may be skewed or incomplete.

Transfer students, who typically enter as juniors, may have further confounded some responses since their unique pathways differ from those of students who started as freshmen. This makes it challenging to isolate trends among traditional juniors. Moreover, there is a clear non-response bias in the dataset, with students performing well academically being more likely to participate, while those struggling were underrepresented. This skews the data toward more positive outcomes and limits the study’s ability to capture the full range of student experiences. Taken altogether, these limitations suggest that conclusions drawn from this study should be interpreted with caution and not generalized to all students without additional, more representative data.

## Conclusion

The financial, social, and academic experiences of Animal Science undergraduates are deeply intertwined, with first-generation and transfer students facing heightened challenges in all domains. This study reinforces the importance of providing robust financial support, cultivating peer networks, and offering proactive academic and career guidance. Drawing on the frameworks of Tinto (1993) and Bandura (2002), these findings highlight that student success is not solely a matter of personal resilience but is shaped by the structures and relationships within the educational environment. To promote impartial student outcomes, institutions must move beyond generalized programming to implement targeted, data-informed supports that address the lived realities of diverse student groups. Initiatives that strengthen peer belonging, improve financial support, and build faculty-student connections will be especially vital in closing success gaps for first-generation and transfer students in the agricultural sciences.

## Data Availability

The data underlying this article cannot be shared publicly due to the privacy of individuals that participated in the study

## Abbreviations

ANS: Animal Science
CALS: College of Agriculture and Life Sciences
CFA: confirmatory factor analysis
DFHS: direct from high school
DYS: Dairy Science
GENPV: General Pre-Veterinary Medicine
GPA: grade point average
IRB: Institutional Review Board
RMSEA: root mean square error of approximation
TLI: Tucker–Lewis Index
